# Differential cytokine/chemokine profiles and reactivation of human herpes viruses in various forms of cutaneous adverse drug reactions. A comparative study in Taiwan

**DOI:** 10.1186/2045-7022-4-S3-P20

**Published:** 2014-07-18

**Authors:** Chia-Yu Chu, Yi-Chun Chen, Che-Wen Yang, Kai-Lung Chen, Yung-Tsu Cho, Chia-Ying Chang, I-Chun Lin

**Affiliations:** 1 Department of Dermatology, National Taiwan University Hospital, Taiwan; 2 National Taiwan University Hospital, Department of Dermatology, Taiwan

## Background

Sequential human herpes virus (HHV) reactivation is well-known in drug reaction with eosinophilia and systemic symptoms (DRESS), but a comparison study to investigate the association of HHV reactivation with other types of cutaneous adverse drug reactions (cADRs) is still lacking. Besides, the pathomechanisms or mediators of viral reactivations are largely unknown. In this study, we aimed to investigate the cytokine/chemokine profiles before or concurrent with HHV reactivations.

## Method

We conducted a prospective study to evaluate HHV-6, Epstein--Barr virus (EBV) and cytomegalovirus (CMV) reactivation rates in various cADRs. Dynamic changes of cytokines and chemokines were also determined by sequential blood tests during acute stages. Interleukin-1£] (IL-1£]), IL-1 receptor antagonist (IL-1Ra), IL-2, IL-4, IL-5, IL-6, IL-7, IL-8, IL-9, IL-10, IL-12, IL-13, IL-15, IL-17, basic fibroblast growth factors, eotaxin, granulocyte colony-stimulating factor, granulocyte-macrophage colony-stimulating factor, interferon-£^ (IFN-£^), interferon £^-induced protein-10 (IP-10), monocyte chemoattractant protein-1, macrophage inflammatory protein-1£\ (MIP-1£\), MIP-1£], platelet-derived growth factor, chemokine (C-C motif) ligand 5 (CCL5), tumor necrosis factor-£\ (TNF-£\) and vascular endothelial growth factor were measured using the Bio-Plex Human Cytokine 27-Plex panel.

## Results

A total of 62 patients were enrolled in this prospective study including 23 DRESS, 17 SJS/TEN, 13 MPE, 5 GBFDE and 4 EMM. HHV-6 reactivation was observed in ten DRESS patients (43.5%) but in none of the other cADR patients. In contrast, EBV reactivation was detected in 35 patients distributed in every cADR groups. EBV reactivation rates were 73.9% for DRESS (17 in 23 patients), 29.4% for SJS/TEN (5 in 17 patients), 53.8% for MPE (7 in 13 patients), 80% for GBFDE (4 in 5 patients), and 50% for EMM (2 in 4 patients). CMV reactivation rates were 43.5% in DRESS (10 in 23 patients) and 11.8% in SJS/TEN (2 in 17 patients) respectively, but none of the other cADRs. HHV-6 reactivation in DRESS patients was associated with decreased proinflammatory cytokines and chemokines (IL-1£], IL-1Ra, IL-2, TNF-£\, IFN-£^, and MIP-1£\) before viral reactivations. Only one chemokine, IP-10, was expressed higher in the HHV-6 reactivation group (P = 0.018).

## Conclusions

HHV-6 reactivation in DRESS was associated with dysregulation of microenvironments mediated by several cytokines and chemokines.

